# An AI deep learning algorithm for detecting pulmonary nodules on ultra-low-dose CT in an emergency setting: a reader study

**DOI:** 10.1186/s41747-024-00518-1

**Published:** 2024-11-20

**Authors:** Inge A. H. van den Berk, Colin Jacobs, Maadrika M. N. P. Kanglie, Onno M. Mets, Miranda Snoeren, Alexander D. Montauban van Swijndregt, Elisabeth M. Taal, Tjitske S. R. van Engelen, Jan M. Prins, Shandra Bipat, Patrick M. M. Bossuyt, Jaap Stoker, The OPTIMACT study group, Colin Jacobs, Miranda Snoeren, Jan M. Prins, Shandra Bipat, Jouke Annema, Ludo F. M. Beenen, Dominique Bekebrede-Kaufman, Inge A. H. van den Berk, Patrick M. M. Bossuyt, Brenda Elzer, Tjitske S. R. van Engelen, Betty Frankemölle, Maarten Groenink, Erwin Hoolwerf, Dorine Hulzebosch, Maadrika M. N. P. Kanglie, Saskia Kolkman, Nick H. J. Lobe, Peter A. Leenhouts, Onno Mets, Melanie A. Monraats, Jan Luitse, Saskia Middeldorp, Alexander Montauban van Swijndregt, Jacqueline Otker, Adrienne van Randen, Milan L. Ridderikhof, Johannes A. Romijn, Maeke J. Scheerder, Antoinet J. N. Schoonderwoerd, Laura J. Schijf, Frank F. Smithuis, Jaap Stoker, Geert J. Streekstra, Elizabeth M. Taal, Glenn de Vries, Maaike J. A. Vogel, Ibtisam Yahya

**Affiliations:** 1 Department of Radiology and Nuclear Medicine, Amsterdam UMC, University of Amsterdam, Amsterdam, The Netherlands; 2 Department of Medical Imaging, Radboud University Medical Center, Nijmegen, The Netherlands; 3 Department of Radiology, Spaarne Gasthuis, Haarlem, The Netherlands; 4 Department of Radiology and Nuclear Medicine, OLVG, Amsterdam, The Netherlands; 5 Division of Infectious Diseases, Department of Internal Medicine, Amsterdam UMC, University of Amsterdam, Amsterdam, The Netherlands; 6 Department of Epidemiology & Data Science, Amsterdam UMC, University of Amsterdam, Amsterdam, The Netherlands; 7 Amsterdam Public Health, Methodology, Amsterdam, The Netherlands; 8 Cancer Center Amsterdam, Imaging and Biomarkers, Amsterdam, The Netherlands; 9 Department of Pulmonology, Amsterdam UMC, University of Amsterdam, Amsterdam, The Netherlands; 10 Patient Ambassador, Longfonds, Amersfoort, The Netherlands; 11 Department of Cardiology, Amsterdam UMC, University of Amsterdam, Amsterdam, The Netherlands; 12 Department of Emergency Care, Amsterdam UMC, University of Amsterdam, Amsterdam, The Netherlands; 13 Department of Vascular Medicine, Amsterdam Cardiovascular Sciences, Amsterdam UMC, University of Amsterdam, Amsterdam, The Netherlands; 14 Division of Endocrinology, Department of Internal Medicine, Amsterdam UMC, University of Amsterdam, Amsterdam, The Netherlands; 15 Department of Radiology and Nuclear Medicine and Department of Biomedical Engineering and Physics, Amsterdam UMC, University of Amsterdam, Amsterdam, The Netherlands

**Keywords:** Artificial intelligence, Emergency service (hospital), Multiple pulmonary nodules, Solitary pulmonary nodules, Tomography (x-ray computed)

## Abstract

**Background:**

To retrospectively assess the added value of an artificial intelligence (AI) algorithm for detecting pulmonary nodules on ultra-low-dose computed tomography (ULDCT) performed at the emergency department (ED).

**Methods:**

In the OPTIMACT trial, 870 patients with suspected nontraumatic pulmonary disease underwent ULDCT. The ED radiologist prospectively read the examinations and reported incidental pulmonary nodules requiring follow-up. All ULDCTs were processed *post hoc* using an AI deep learning software marking pulmonary nodules ≥ 6 mm. Three chest radiologists independently reviewed the subset of ULDCTs with either prospectively detected incidental nodules in 35/870 patients or AI marks in 458/870 patients; findings scored as nodules by at least two chest radiologists were used as true positive reference standard. Proportions of true and false positives were compared.

**Results:**

During the OPTIMACT study, 59 incidental pulmonary nodules requiring follow-up were prospectively reported. In the current analysis, 18/59 (30.5%) nodules were scored as true positive while 104/1,862 (5.6%) AI marks in 84/870 patients (9.7%) were scored as true positive. Overall, 5.8 times more (104 *versus* 18) true positive pulmonary nodules were detected with the use of AI, at the expense of 42.9 times more (1,758 *versus* 41) false positives. There was a median number of 1 (IQR: 0–2) AI mark per ULDCT.

**Conclusion:**

The use of AI on ULDCT in patients suspected of pulmonary disease in an emergency setting results in the detection of many more incidental pulmonary nodules requiring follow-up (5.8×) with a high trade-off in terms of false positives (42.9×).

**Relevance statement:**

AI aids in the detection of incidental pulmonary nodules that require follow-up at chest-CT, aiding early pulmonary cancer detection but also results in an increase of false positive results that are mainly clustered in patients with major abnormalities.

**Trial registration:**

The OPTIMACT trial was registered on 6 December 2016 in the National Trial Register (number NTR6163) (onderzoekmetmensen.nl).

**Key Points:**

- An AI deep learning algorithm was tested on 870 ULDCT examinations acquired in the ED.
- AI detected 5.8 times more pulmonary nodules requiring follow-up (true positives).
- AI resulted in the detection of 42.9 times more false positive results, clustered in patients with major abnormalities.
- AI in the ED setting may aid in early pulmonary cancer detection with a high trade-off in terms of false positives.

**Graphical Abstract:**

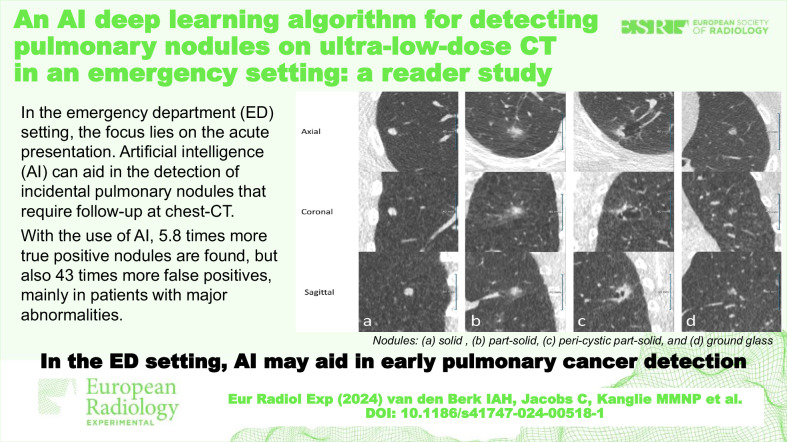

## Background

Incidental findings at imaging are common and are usually defined as abnormalities not related to the indication for performing the imaging examination [1]. At chest computed tomography (CT), incidental pulmonary nodules are the most frequently found incidental findings [2, 3]. Appropriate management of these nodules has been clearly defined [4, 5]. With the increasing use of CT scans in the emergency department (ED) setting, the ability to also accurately detect incidental pulmonary nodules is important for early pulmonary cancer detection.

To our knowledge, there are four previous studies reporting on incidental pulmonary nodules on chest CT at the ED, three of them being retrospective studies on CT pulmonary angiography [6–8] and one prospective study on low-dose CT in elderly patients suspected of pneumonia [9]. Those studies reported incidental pulmonary nodules that require follow-up in a range between 8.3% and 9.9% of patients [6–9], as compared to 19.7% in the first round of the Nelson pulmonary cancer screening trial [10]. No studies on ultra-low-dose CT (ULDCT) are available.

We recently reported the results of the “OPTimal IMAging strategy in patients suspected of non-traumatic pulmonary disease at the ED: chest x-ray or CT” (OPTIMACT) trial, a randomized controlled clinical trial that evaluated the effect on health outcomes of replacing chest x-ray (CXR) by ULDCT in the diagnostic work-up of patients suspected of non-traumatic pulmonary disease at the ED [11]. In the OPTIMACT trial, as expected, there were more incidental pulmonary nodules that required follow-up in the ULDCT-arm compared to the CXR-arm of the trial: 54/1,200 (4.5%) *versus* 7/1,203 (0.6%), respectively. The number of incidental pulmonary nodules that required follow-up in the ULDCT arm of the trial was lower than reported in the aforementioned earlier studies [6–9]. This might be explained by the difference in patient population, namely: the OPTIMACT trial included patients suspected of non-traumatic pulmonary disease requiring CXR, *versus* elderly patients suspected of pneumonia or patients suspected of pulmonary emboli.

However, in the hectic work environment of the ED the primary focus lies on the acute problem of presentation; the focus on relevant incidental findings may be lower compared to the outpatient setting. It is known that computer-aided pulmonary nodule detection can aid in the detection of pulmonary nodules in the ED setting [12]. In recent years, the advent of artificial intelligence (AI) deep learning algorithms has improved the detection of pulmonary nodules even further [13]. AI deep learning-based algorithms for pulmonary nodule detection have been primarily developed for regular and low-dose chest CT, but recent studies also reported good performance for pulmonary nodule detection on ULDCT [14–16].

We hypothesised that AI for pulmonary nodule detection on ULDCT in the ED setting would result in improved detection of incidental pulmonary nodules that require follow-up. The primary objective of this study was to investigate this added value of an AI algorithm, *i.e.*, its impact in terms of finding additional pulmonary nodules that require follow-up. Secondary objectives were to investigate the probability of malignancy of those nodules, and to assess the performance of the AI software on ULDCT images with respect to the number of true and false positives.

## Methods

### Study design

The Medical Ethics Committee approved the study protocol of the OPTIMACT trial. All participants provided written informed consent. The OPTIMACT trial was registered in the Trial Register (number NTR6163). For this retrospective secondary analysis, a waiver was obtained.

This study is an additional analysis of data collected in the OPTIMACT trial [11, 17]. In that trial, which ran between January 31, 2017 and May 31, 2018, during randomly assigned periods of one calendar month either ULDCT or CXR was used as the radiological imaging modality in patients who required pulmonary imaging at the ED of two participating Dutch hospitals: one university hospital and one large teaching hospital [17].

### Study participants

The OPTIMACT study included 2,418 patients aged 18 years and older, presenting at the ED suspected of non-traumatic pulmonary disease and requiring chest imaging according to the attending physician. Excluded patients were those who were unable to undergo ULDCT or CXR, pregnant women, incapacitated patients, and patients with a life expectancy of less than one month or with other anticipated barriers to 28 days of follow-up data collection. Patients could only participate once. Further details have been reported elsewhere [11, 17]. The OPTIMACT trial was designed as a multicentre trial to make the results of our trial applicable for a larger population. For practical reasons only trial participants of the ULDCT arm of the university hospital (*n* = 870) were included in this substudy.

### Study procedures

All ULDCTs were performed using a Somatom Force scanner installed in 2015 (Siemens Healthineers, Erlangen, Germany) with fixed 100 kVp, Sn filter, reference 50 mAs, collimation 192 × 0.6 mm, lung window (width 1,600 UH; level -500 UH), slice reconstruction thickness 1 mm, increment 1 mm, kernel BL57, iterative reconstruction advanced modelled iterative reconstruction—Admire 3. The median dose of the ULDCTs was 0.24 mSv (interquartile range [IQR]: 0.19–0.31 mSv) with a volumetric CT dose index of 0.41 ± 0.21 mGy (mean ± standard deviation), sufficient for the detection and evaluation of solid and sub-solid pulmonary nodules [14].

All ULDCTs were read prospectively on a picture archiving and communication system (Enterprise Imaging, v8.1.4, AGFA Health Care, Mortsel, Belgium) by either radiology residents (28 radiology residents with years of experience in reading chest CT ranging 1–5, mean ± standard deviation 3.4 ± 1.2) under supervision of a radiologist or by the ED radiologist (18 radiologists with years of experience in reading chest CT ranging 5–19, mean ± standard deviation 10.4 ± 3.7) at the time of clinical management, also outside office hours. As all examinations were either read or supervised by a radiologist, who bears the ultimate responsibility, we consider these examinations as read by the ED radiologist. Following routine clinical practice, radiologists had full access to the patients medical record. There was a previous chest CT examination available in 320/870 (36.8%) of patients. During the trial, structured standardized reading and reporting was performed by the ED radiologist, including the main diagnosis and the presence of clinically relevant incidental findings [17]. For the evaluation of incidental pulmonary nodules, the 2017 Fleischner Society guideline [4] was used.

### AI algorithm

In this study we used an academic deep learning-based pulmonary nodule detection AI algorithm, the DIAG Lung Nodule AI, v2.9 (Diagnostic Image Analysis Group, Radboud University Medical Center, the Netherlands), that detects solid, part-solid and non-solid pulmonary nodules with a diameter of 4 mm or larger. The details of the AI software and the performance have been previously reported [18]. The AI algorithm uses a multi-view deep learning architecture that analyses multiple two-dimensional cross-sectional images through the nodule and has been trained on a large set of 888 cases from the publicly available LUNA16 database. The AI software produces a list of AI marks, and for each mark, nodule type, estimated diameter, major axis, and volume is provided [18].

### Reference standard and reader study

To establish the reference standard, three chest radiologists evaluated all ULDCTs of the patients with incidental pulmonary nodules that require follow-up prospectively detected by the radiologist during ED reading (“ED nodules”) or with *post hoc* AI marks. These chest radiologists (OMM, MS, and ADMvS) had 9 years, 19 years, and 28 years of experience in reading chest CT studies, respectively. All readers were experienced in using the 2017 Fleischner Society guidelines for the evaluation of pulmonary nodules in clinical practice [4] and were familiar with the morphology of perifissural nodules (PFNs) described in the literature [19, 20]. Guidance was provided in the form of a step-by-step plan listing the procedure to be followed and the definition of morphologic features of pulmonary nodules; the step-by-step-plan is available in the Supplementary Material.

**Fig. 1:**
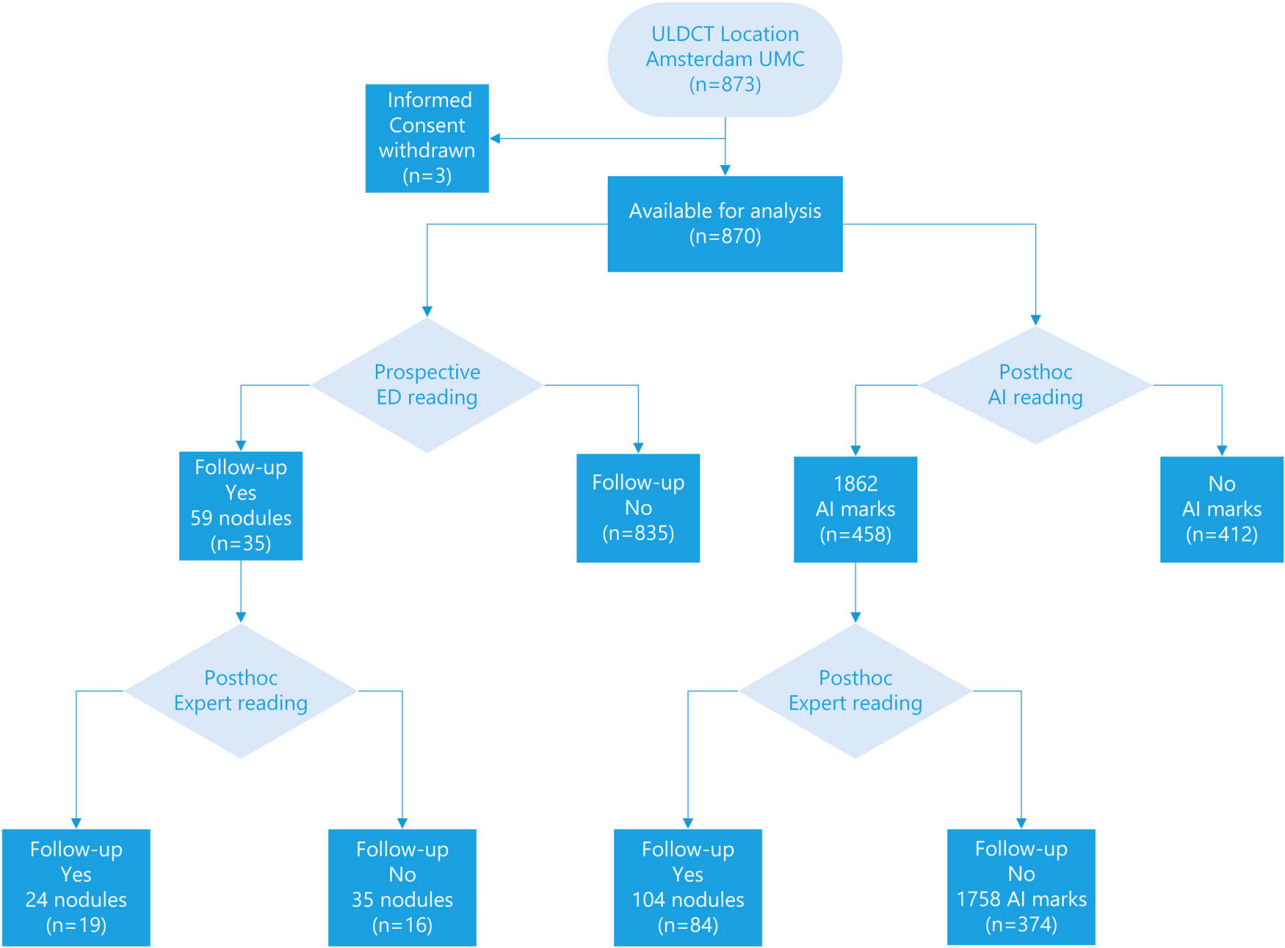
Study flow chart. AI, Artificial intelligence; ED, Emergency department; ULDCT, Ultralow-dose computed tomography

These experts performed expert readings on the two data sets using a dedicated reading workstation (CIRRUS Lung Screening, v19.9.2, Diagnostic Image Analysis Group, Radboud University Medical Center, the Netherlands). CT scans were independently read, all readers were blinded to patient information, the post-imaging outcomes and each other’s results. Previous imaging was not available. The expert readers were blinded for the incidental pulmonary nodules found by the radiologist during ED reading but aware that this radiologist had identified one or more incidental pulmonary nodules that required follow-up. The reading workflow is depicted in Fig. 1.

First, the expert readers evaluated the ULDCTs with ED nodules (*n* = 35). Subsequently, after an interval of four weeks, they independently evaluated all AI marks for noncalcified nodules ≥ 6 mm [21]. This simulated a second expert reading of the results of the AI system, as those results would have been presented to the ED radiologist if the AI system was available during the trial. For this analysis, we lowered the size threshold of the AI software for detection to a diameter ≥ 6 mm, in line with the 2017 Fleischner Society criteria [4].

**Fig. 2:**
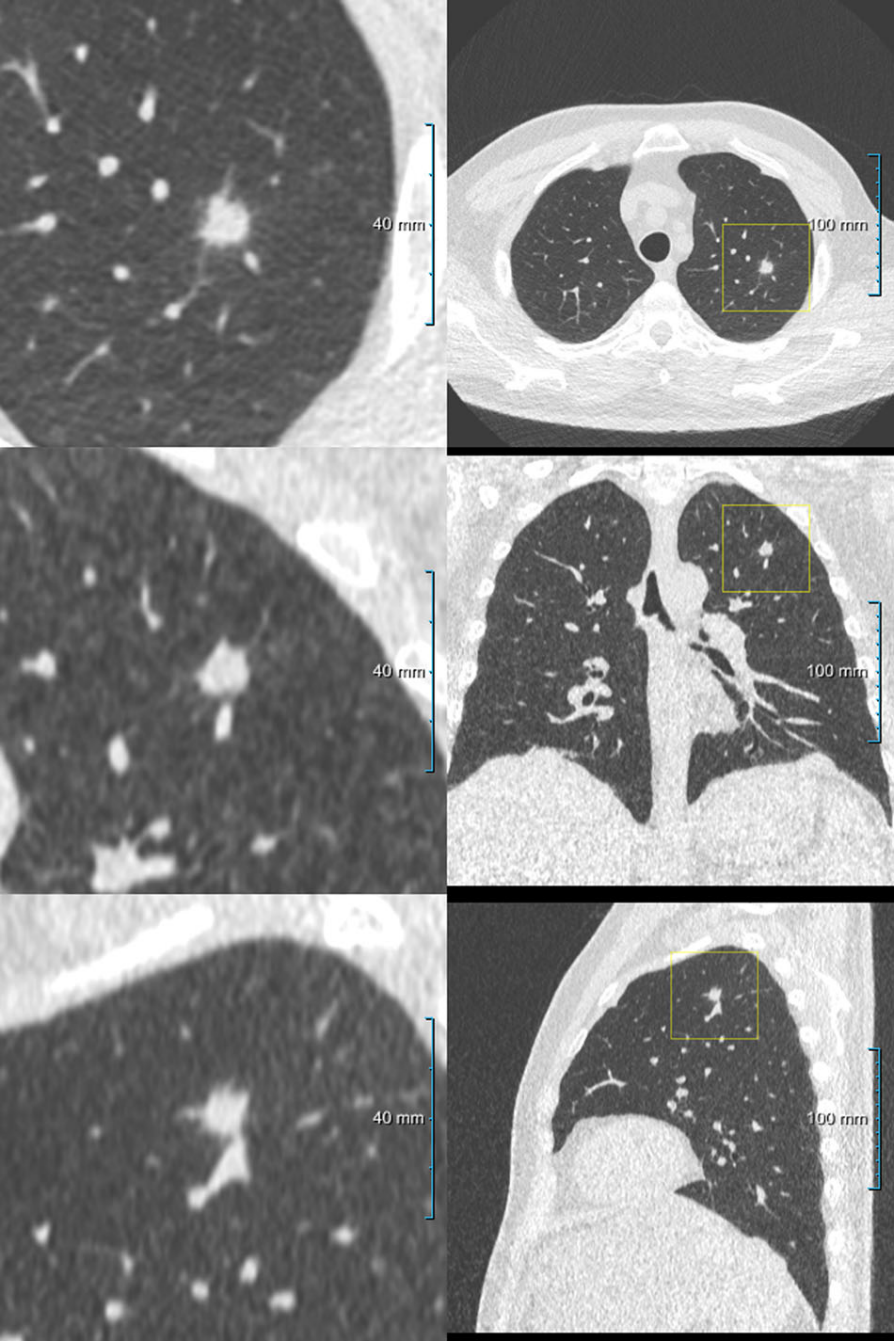
A spiculated solid pulmonary nodule in the left upper lobe visualized at CIRRUS reading workstation

The reading workstation enabled image viewing in different window/level settings and provided reconstructions in all three orthogonal planes (Fig. 2). After annotation of the lung nodule estimated diameter, major axis, and volume were measured by the workstation. Nodule type and location of the nodule in the lung were scored for each annotated pulmonary nodule by the expert radiologist. Noncalcified pulmonary nodules were classified as solid, part-solid, or non-solid nodules (ground glass). In case of solid nodules with sharp margins a distinction was made between PFNs and non-PFN nodules, based on: shape (round, oval, or polygonal), distance from the pleura (< 15 mm), and diameter (< 12 mm) [19, 20]. Calcified nodules were considered benign, not requiring follow-up, and were therefore disregarded [22]. In addition to nodule morphology, the presence of spiculation was scored for each annotated nodule. When the experts in majority did not agree on the characteristics of a nodule, the nodule characteristic was scored as inconclusive.

Besides the nodule characteristics, the presence or absence of emphysema was visually assessed. To define a pulmonary nodule that requires follow-up, we used the 2017 Fleischner Society guidelines for the high-risk group but included patients under 35 years and with a history of malignancy [4]. We considered incidental pulmonary nodules requiring follow-up that were scored by at least two readers as the reference standard for “true positives”.

### Outcomes

The primary study outcome was the proportions of incidental pulmonary nodules that require follow-up based on the 2017 Fleischner Society guideline after expert reading of both the 35 ULDCTs with ED nodules and the AI marks [4]. Secondary outcomes were to predict the probability of malignancy by calculating the median adjusted Brock risk score of the true positive incidental pulmonary nodules in both groups [23] and to assess the performance of the AI software on the ULDCT images. The Brock risk score estimates the probability that a lung nodule will be diagnosed as cancer within a 2- to 4-year follow-up period [23]. An adjusted Brock risk score was calculated because there was no information available on the family history of lung cancer and total nodule count on CT.

### Statistical analysis

Continuous data were reported as means and standard deviations or medians and IQRs, categorical data as numbers and percentages.

True positives were defined as those incidental pulmonary nodules requiring follow-up as confirmed at expert reading. False positives were defined as those incidental pulmonary nodules not requiring follow-up according to the expert reading. The proportion true positive results of the ED reading was calculated by comparing the number of ED nodules that required follow-up after expert reading with the total number of ED nodules. The proportion of true positive results of the AI marks was calculated by comparing the AI marks identified as incidental pulmonary nodules that required follow-up after expert reading with the total number of AI marks. Both the ED nodules and the AI marks that were not scored by at least two expert readers as an incidental pulmonary nodule that requires follow-up were considered false positives. The difference in proportion of the true positive and false positive results of the ED nodules and AI marks were calculated. We calculated the median adjusted Brock score of the true positive results of both strategies.

The performance of the AI software was assessed by comparing the true and false positive ED nodules with the true and false positive AI marks and by calculating the median number of AI marks per ULDCT.

Differences between groups were analysed with two-tailed χ^2^ or Fisher exact for categorical data and by the Mann–Whitney *U*-test for continues data, since these had no normal distribution. We considered *p*-values lower than 0.05 as significant.

All analyses were performed in IBM SPSS Statistics for Windows, version 26 (IBM Corp., Armonk, New York, USA).

## Results

The 870 participants of the ULDCT arm of the OPTIMACT trial scanned at the university hospital were included in this retrospective analysis. Baseline characteristics of the patients are available in Table 1.

**Table 1:** Baseline characteristics of study participants (*n* = 870)

Demographics	
Age, years (mean ± standard deviation)	56.9 ± 18.5
Female sex	435 (50.0)
Smoking history	
Active smoker	138 (15.9)
Former smoker	273 (31.4)
Non-smoker	416 (47.8)
Smoking history unknown	43 (4.9)
Comorbidity	
Immunocompromised	240 (27.5)
Malignancy	179 (20.6)
Diabetes	178 (20.5)
Pulmonary disease	235 (27.0)
Chronic obstructive pulmonary disease	116 (13.3)
Asthma	105 (12.1)
Interstitial lung disease	22 (2.5)
Cystic fibrosis	14 (1.6)
Cardiac disease	166 (19.1)
Myocardial infarction	118 (13.6)
Chronic cardiac failure	77 (8.9)
Neurological disease	99 (11.4)
Kidney disease	83 (9.5)
Thromboembolic disease	67 (7.7)

Data are given as absolute numbers (percentages) with the only exception of age, given as mean ± standard deviation

### Prospective reading by the ED radiologist compared to expert reading

During the primary reading by the ED radiologist, pulmonary nodules were reported for 236 of the 870 ULDCTs (27.1%), 129/870 (14.8%) with solitary pulmonary nodules, 107/870 (12.3%) with multiple pulmonary nodules. On 35/870 (4.0%) of the ULDCTs, the ED radiologist reported incidental nodules that required follow-up (“ED nodules”) for a total of 59 ED nodules. After expert reading of these 35 ULDCT examinations, 24 incidental pulmonary nodules that required follow-up (true positive) were found in 19 ULDCTs (19/870, 2.2%) (Fig. 1). Six of these 24 pulmonary nodules (25.0%) were newly detected pulmonary nodules that require follow up during expert reading; 16 of these 24 pulmonary nodules (66.7%) were detected by the AI system, 8 pulmonary nodules were not detected by the AI system (33.3%). The proportion of true positives over false positives at the primary reading by the ED radiologist was 0.44% (18/41).

### AI algorithm compared to expert reading

The *post hoc* processing of all 870 ULDCT scans using the AI software resulted into 1,862 AI marks in 458/870 ULDCTs (52.6%). After expert reading, 104 AI marks in 84/870 ULDCTs (9.7%) were considered incidental pulmonary nodules that required follow-up by at least two of the three expert chest radiologists (Fig. 1). The proportion of true positives over false positives was 0.06 (104/1,758).

### Prospective reading by the ED radiologist *versus* AI algorithm

**Fig. 3:**
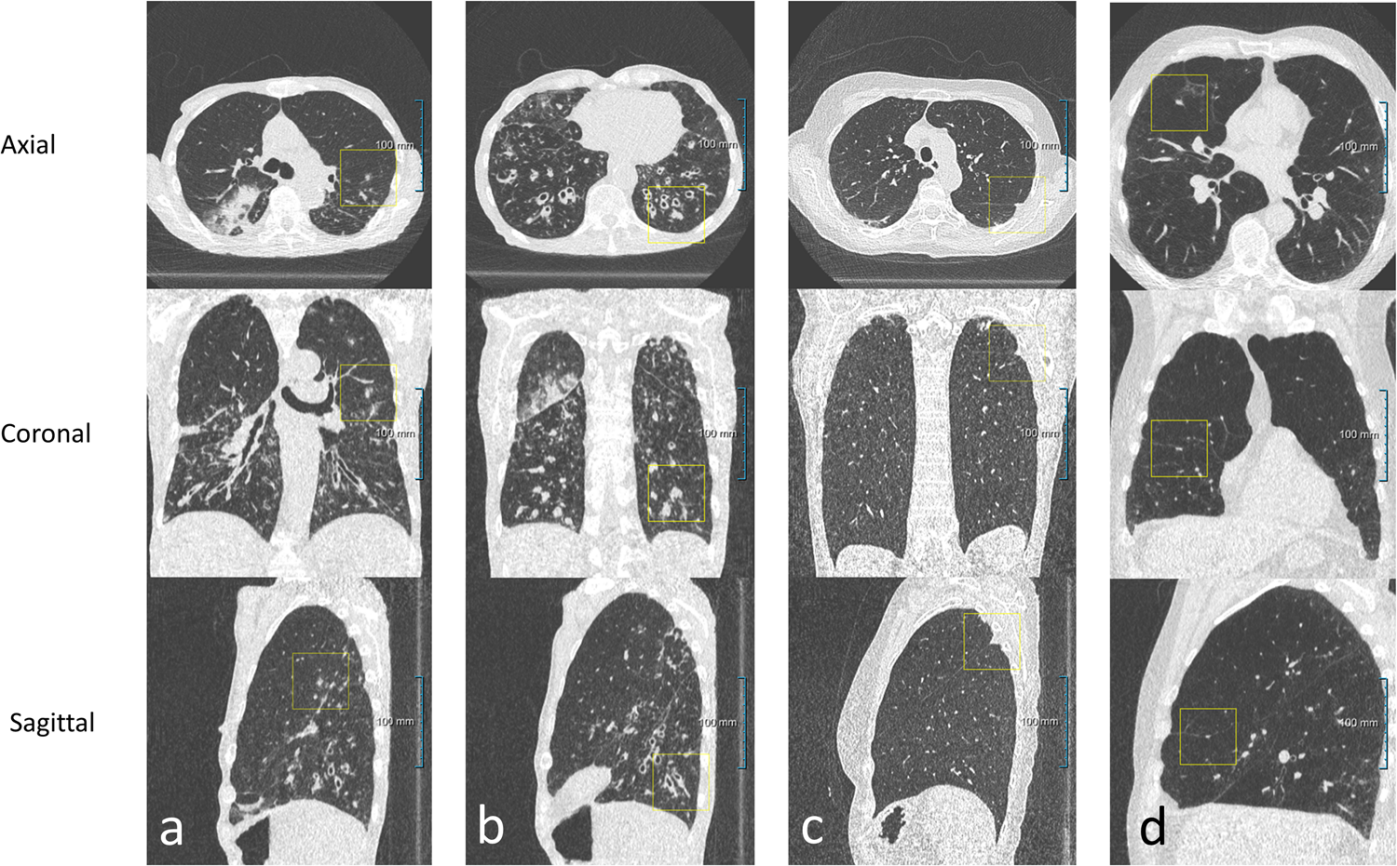
Multiplanar projections of various false positive results: (**a**) an AI-marked part-solid nodule, being part of a bronchopneumonia; (**b**) an AI-marked solid nodule, being a mucus impaction in a bronchiectasis; (**c**) an AI-marked solid nodule, being a focal interfissural pleural thickening; and (**d**) an AI-marked solid nodule, being a solid nodule that requires follow-up at the prospective reading by the ED radiologist, being a PFNs that does not require follow-up

The expert reading of the ULDCTs with ED nodules resulted in a lower absolute number, but a higher proportion of true positives compared to the expert reading of the AI marks, 18/59 (30.5%) *versus* 104/1,862 (5.6%) (*p* < 0.001, two-tailed χ^2^). The expert reading of the ULDCTs with ED nodules resulted in a lower number and a lower proportion of false positives compared to the expert reading of AI marks, 41/59 (69.5%) *versus* 1,758/1,862 (94.4%) (*p* < 0.001, two-tailed χ^2^). With the AI software 5.8 times (104/18) more true positives were found and 42.9 (1,758/41) times more false positives. Examples of various false positive results are given in Fig. 3.

### Pulmonary nodule characteristics and adjusted Brock risk score

**Fig. 4:**
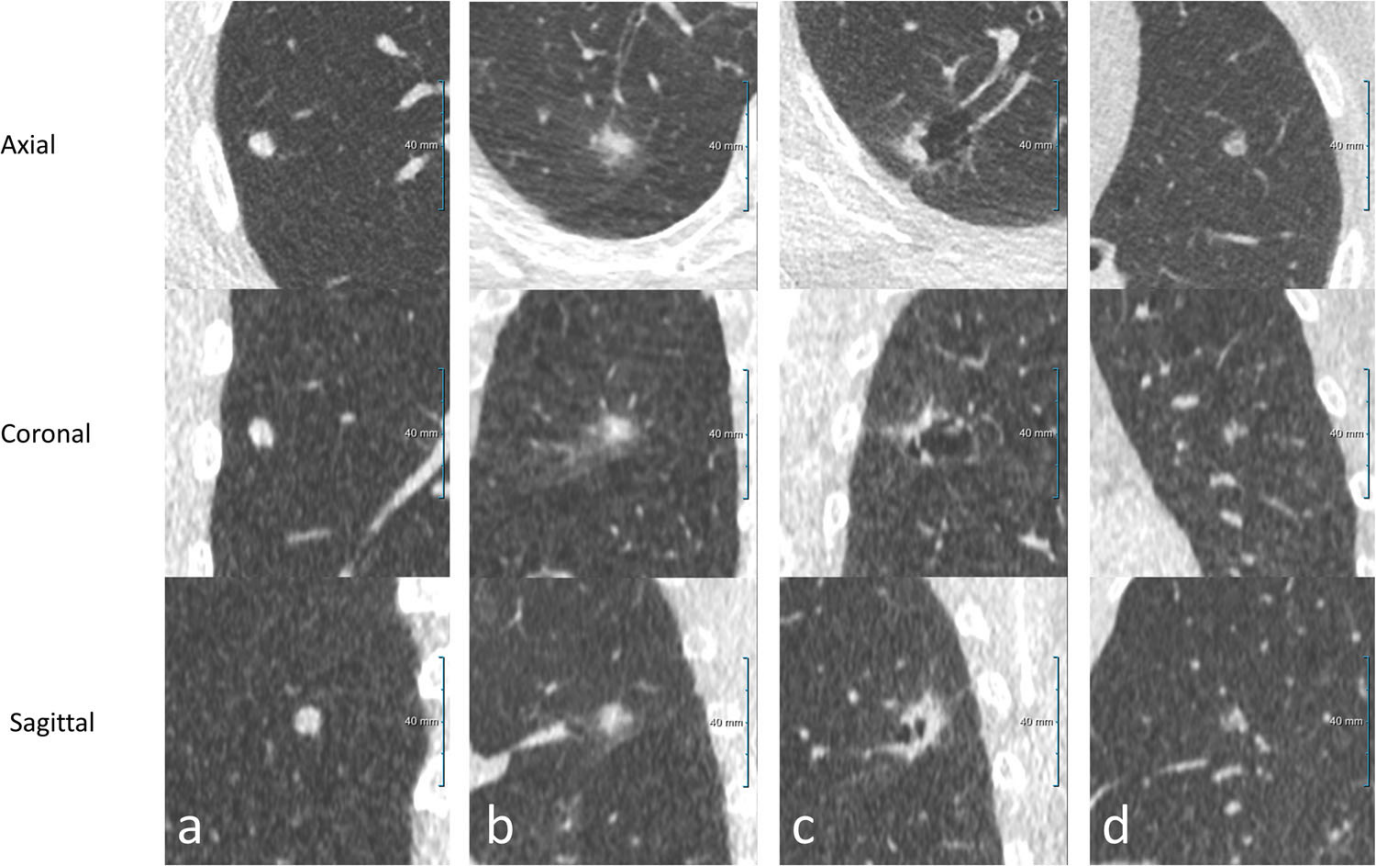
Multiplanar projections of various incidental pulmonary nodules: (**a**) solid nodule at the right lower lobe; (**b**) part-solid nodule at the right upper lobe; (**c**) peri cystic part-solid nodule at the right upper lobe; and (**d**) non-solid (ground glass) nodule at the lingula left upper lobe

ED nodules were larger in volume compared to the AI marks, with a median of 450.8 mm^3^ (IQR: 145.0–1,235.8) *versus* 289.0 mm^3^ (IQR: 173.3–547.1) for the AI marks, though not significant different (*p* = 0.083), Mann–Whitney *U*-test. Further imaging characteristics of the true positives of the ULDCTs with ED nodules and of the AI marks are presented in Table 2 and Fig. 4. The median adjusted Brock risk score was 10.3% (IQR: 5.5–32.1) for the 24 true positives in the ULDCTs with ED nodules and 6.1% (IQR: 2.4–12.6) for the 104 true positive AI marks (*p* = 0.035), Mann–Whitney *U*-test.

**Table 2:** Characteristics of true positive incidental pulmonary nodules requiring follow-up

Classification	ED reading, (*n* = 24)	AI marks, (*n* = 104)	*p*-value^*^
Solid	19 (79)	74 (71)	0.427
Part-solid	1 (4)	8 (8)	1.000
Non-solid	2 (8)	14 (13)	0.735
Inconclusive	2 (8)	8 (8)	1.000
Size			
Diameter, mm (median IQR)	9.4 (7.5–13.3)	8.1 (6.9–10.1)	0.085
Major axis, mm (median IQR)	12.1 (9.5–20.2)	9.9 (8.7–13.1)	0.058
Volume, mm^3^ (median IQR)	450.8 (145.0–1235.8)	289.0 (173.3–547.1)	0.083
Aspect			
Spiculation	0 (0)	2 (2)	1.000
Spiculation inconclusive	0 (0)	1 (1)	1.000
Presence of emphysema	9 (38)	21 (20)	0.712
Emphysema inconclusive	3 (13)	9 (9)	0.696
Location			
Right upper lobe	11 (46)	34 (33)	0.224
Middle lobe	3 (13)	5 (5)	0.170
Right lower lobe	5 (21)	20 (19)	0.858
Left upper lobe	4 (17)	24 (23)	0.593
Left lower lobe	1 (5)	20 (19)	0.122
Lobe inconclusive	0 (0)	1 (1)	1.000

True positives pulmonary nodules were defined as those incidental pulmonary nodules requiring follow-up as confirmed at expert reading

Data are absolute numbers (percentages) unless otherwise specified, two-tailed χ^2^ or Fisher exact test (*n* < 5) was used for categorical data, the Mann–Whitney *U*-test for continues data accounting for the non-normal distribution of the continues data

*AI* Artificial intelligence, *ED* Emergency department, *IQR* Interquartile range

### The performance of AI software on ULDCT images

**Fig. 5:**
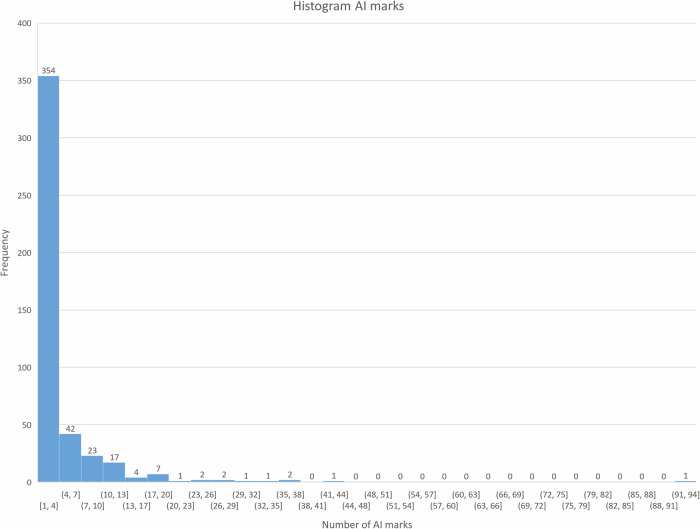
Histogram distribution of AI marks. In 354 of the 458 ULDCTs there were 1–4 AI marks; only 22 patients had more than 13 AI marks. AI, Artificial intelligence; ULDCT, Ultralow-dose computed tomography

The median number of AI marks across the full set of 870 ULDCTs was 1 (IQR: 0–2) per ULDCT. The median number of AI marks across the set of 458 ULDCTs with AI marks was 2 (IQR: 1–4). There were 10 patients with 20 or more AI marks with a maximum of 92 AI marks. There were 44 patients with 10 or more AI marks. A histogram of the AI marks is presented in Fig. 5.

## Discussion

The use of AI software on ULDCT scans of patients suspected of nontraumatic pulmonary disease at the ED resulted in the detection of 5.8 times more incidental pulmonary nodules that require follow-up than reported by the ED radiologist, but also resulted in 42.9 times more false positive results. Nodules detected by the AI system had a significantly lower adjusted Brock risk score.

ULDCTs made in the ED setting included patients with major lung abnormalities such as pneumonia or lung metastases. False positive AI marks were clustered in those patients reflected by the high number of AI marks (1,862/458) but low overall median number of 1 (IQR: 0–2) AI marks per ULDCT. In most patients with major lung abnormalities, the burden of false positive results for the radiologist is limited because the appearance of, for example a bronchopneumonia pattern, is clear instantly and does not need scrutiny of all false positive results. Still for an efficient workflow, in an often-demanding ED environment, the false positive rate needs to be reduced. This can be done by optimizing an AI algorithm for the detection of pulmonary nodules requiring follow-up specifically for ULDCT acquired in an ED setting. Further development of AI systems in nodule characterisation will improve cancer risk prediction of incidental pulmonary nodules contributing to a further reduction in false positive results [24].

The ED radiologists detected 18 out of the 24 (75.0%) true positive incidental pulmonary nodules requiring follow-up during the ED reading. During the *post hoc* AI reading, a total of 104 true positive incidental pulmonary nodules requiring follow-up were detected. The true positive results in this study were defined as those incidental pulmonary nodules requiring follow-up as confirmed at expert reading and does not represent the true prevalence of pulmonary nodules in the study population.

The increased detection of pulmonary nodules by an AI algorithm is in line with two prospective studies in the outpatient setting that showed an increase in the sensitivity of nodule detection by the radiologist with the use of computer aided diagnosis, the increase in sensitivity was however reported as variable between radiologists [14, 25]. The decrease in the number of incidental pulmonary nodules that require follow-up after expert reading is most likely related to the variable application of the 2017 Fleischner Society guidelines by radiologists in general [26] and the confidence to classify nodules as benign PFN. Because of the higher signal- to-noise ratio on ULDCT, the expert radiologist experienced the classification of PFN as more difficult compared to regular dose CT.

The median Brock score of the true positive results of the ULDCTs with ED nodules was significantly higher (*p* = 0.035) compared to the true positive AI marks, 10.3% *versus* 6.1% probability of malignancy, respectively. The difference is partly related to the (non-significant) difference in size of the nodules, the median volume of the nodules was 451 mm^3^ *versus* 289 mm^3^, respectively. This implicates that AI especially can help in the detection of smaller incidental pulmonary nodules with clinical relevance, as follow-up is advised with a Brock score > 0% and < 10% [27].

Because simulation of the ED practice with concurrent or second reading of the AI marks was not possible, we adopted a *post hoc* AI analysis with second expert reading of the AI marks, as the 1,862 AI marks would have been presented to the ED radiologist if the system would have been available during the trial [21]. Our primary aim was to investigate the added value of the AI system in an ED setting. The assessment of the performance of DIAG Lung AI software on ULDCT images is hampered by this approach.

This study has limitations. First, the absence of previous imaging during the expert reading. This may have influenced the number of nodules requiring follow-up after expert reading, since growth of a nodule or the appearance of a new nodule is very important for risk stratification [4, 5]. Second, the 28-day follow-up period prohibited the investigation of the number of pulmonary malignancies found during long-term follow-up. Third, we performed expert reading of the ULDCTs with AI marks only (458/870, 52.6%) ULDCTs) and not an expert reading of the full data set. Therefore, assessment of the sensitivity and false negative proportion of the DIAG Nodule AI software could not be performed.

A strength of our study is the prospective recruitment of study participants. ULDCT was routinely used in consecutive patients suspected of pulmonary disease with an indication for chest imaging, presenting at the ED of a tertiary academic hospital. The results are representative of an urban population and generalizable to other urban settings.

In conclusion, the use of AI on ULDCT in ED patients suspected of nontraumatic pulmonary disease results in the detection of more incidental pulmonary nodules that require follow-up but is still limited by a high false positive rate. The impact of AI on the detection of early lung cancer needs further research in the form of a prospective study with long-term follow-up.

## Supplementary information

ELECTRONIC SUPPLEMENTARY MATERIAL

## Data Availability

The datasets are available from the corresponding author on reasonable request.

## Abbreviations

AI: Artificial intelligence
CT: Computed tomography
CXR: Chest x-ray
ED: Emergency department
IQR: Interquartile range
OPTIMACT: OPTimal IMAging strategy in patients suspected of non-traumatic pulmonary disease at the Emergency Department: chest x-ray or CT
PFN: Perifissural nodule
ULDCT: Ultralow-dose computed tomography
