# Supplementary material for: An AI deep learning algorithm for detecting pulmonary nodules on ultra-low-dose CT in an emergency setting: a reader study
Source: Eur Radiol Exp. 2024 Nov 20;8:132. doi: 10.1186/s41747-024-00518-1 (PMC11579269; doi:10.1186/s41747-024-00518-1)
Supplement: Supplementary file 1 — ELECTRONIC SUPPLEMENTARY MATERIAL [file 41747_2024_518_MOESM1_ESM.docx]

# Supplementary material

# Van den Berk IAH, Jacobs C, Kanglie MMNP et al. OPTIMACT study group. An AI deep learning algorithm for detecting pulmonary nodules on ultra-low-dose CT in an emergency setting: a reader study

# Guidance information Chest Radiologists

# Step-by-Step Plan: AI Lung Nodule Sub-study

## Reading Clinically Found Nodules

**Archive: OPTIMACT-clin-nodes-1**

Regarding the assessment of clinically found nodules, we have agreed to proceed as follows:

- You know that there are clinically found nodules on these scans, but you are blinded to the actual findings.
- As experts, assess the scans for the presence of clinically found nodules, considering high-risk patients according to the Fleischner 2017 guideline. This means there is no lower limit. Nodules < 6 mm are scored if suspicious.
- Further score nodules as with AI reading: characterise the nodule, indicate the presence of emphysema.

## AI Reading

**Archive: OPTIMACT-AI**

**Reading: Assess AI results**

1. Does the AI mark correspond to a nodule?
   - If No (in case of vascular structure): Remove AI mark
   - If Yes:
2. Is it an incidental pulmonary lung nodule? Or is it another disease (bronchopneumonia, metastatic malignancy)? Please mention this in the comment of the Report tab. Nodules in the context of another disease do not need to be individually assessed. By not clicking on the results, they do not turn green and are not included in the analysis. It is only possible to place comments regarding the nodule assessment in the Viewer tab.
3. If it is an incidental pulmonary lung nodule, is the segmentation correct? Adjust if necessary.
4. Characterise the nodule:
   - Solid
   - Part-Solid
   - Non-Solid (ground glass)
   - Perifissural (see Schreuder's definition [1])
5. Is spiculation present?
6. Score the lobe the nodule is located in, e.g. RUL, RML, RLL, LUL, LL
7. Clinical relevance of the nodule and risk assessment is done by software. Select all incidental pulmonary nodules, both clinically relevant and clinically not relevant (PFN or smaller than 5mm, 80 mm³).
8. Is emphysema present? Select No or Moderate. The degree of emphysema is not important.

**CIRRUS AI**

- CIRRUS software has been extensively validated but not on ULDCT.
- The software is designed to detect lung nodules between 4 and 30 mm up to a number of 20 lung nodules. The OPTIMACT population contains relatively many patients with bronchopneumonia’s, these patients have > 20 lung nodules, with more than 20 lung nodules, the software skips nodules.
- Scoring the 35 patients with clinically found lung nodules, without AI, comparing with the found AI results provides a measure of the sensitivity of the CIRRUS software on ULDCT.

## Definitions

**Clinically Relevant Lung Nodule:** Lung nodule that requires follow-up according to Fleischner 2017 guideline. Clinical relevance is assessed by CIRRUS software.

**Perifissural Lung Nodule**

Definition according to Schreuder review:

- Non-calcified solid
- Sharp Margins
- Shape: round, oval, polygonal
- Distance ≤ 15 mm from the pleura
- Diameter ≤ 12mm

## AI CIRRUS Software

You can access the software by going to the web address provided in Chrome:

**Archives**

- OPTIMACT-25 (test)
- OPTIMACT-870 (AI): total AMC OPTIMACT population
- OPTIMACT-clin-nodi-1: 35 patients with clinically relevant nodules during ED reading
- OPTIMACT-ai (AI): AMC OPTIMACT population with positive AI result

Select the correct archive, double-click on the desired patient, after approval the next patient appears.

**Viewer**

**Patient Name:**

Display: focus, invert, mirror, reset

**Setting Reconstruction Options:**

- Maximum (MIP) Reconstruction Thickness
- Minimum (MinIP)
- Average (regular)

Adjust window settings: Maximum and minimum window setting

Orientation: O A C S

If necessary, adjust reconstruction thickness to 5 mm average for assessing emphysema.

**Nodule Assessment:**

AI results/selected nodules Delete Type: (volume calculation adjusted to nodule type) Spiculated: click if present

- Solid
- Part-Solid
- Non-Solid (ground glass)
- Calcified (n/a)
- Perifissural
- Endobronchial (n/a)

Lobe: Select lobe

Lesion/Core: adjust if desired

- Threshold:
- Regularity:

Comment: Provide an explanation of the nodule (e.g., guidelines n/a, known metastatic malignancy, nodules part of bronchopneumonia)

**Additional Characteristics**

- Emphysema: only presence or absence
- Coronary Artery Calcification (n/a)
- Airway Wall Thickening (n/a)
- Lymph node involvement (n/a)

**Report**

In the top right corner, select 'Sign off and go to next'.

Let me know if you need any further assistance!

## Relevant figures literature

## PFN [1]


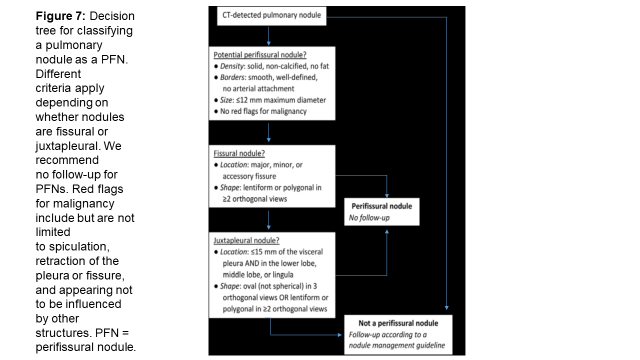


## Fleischner 2017 [2]


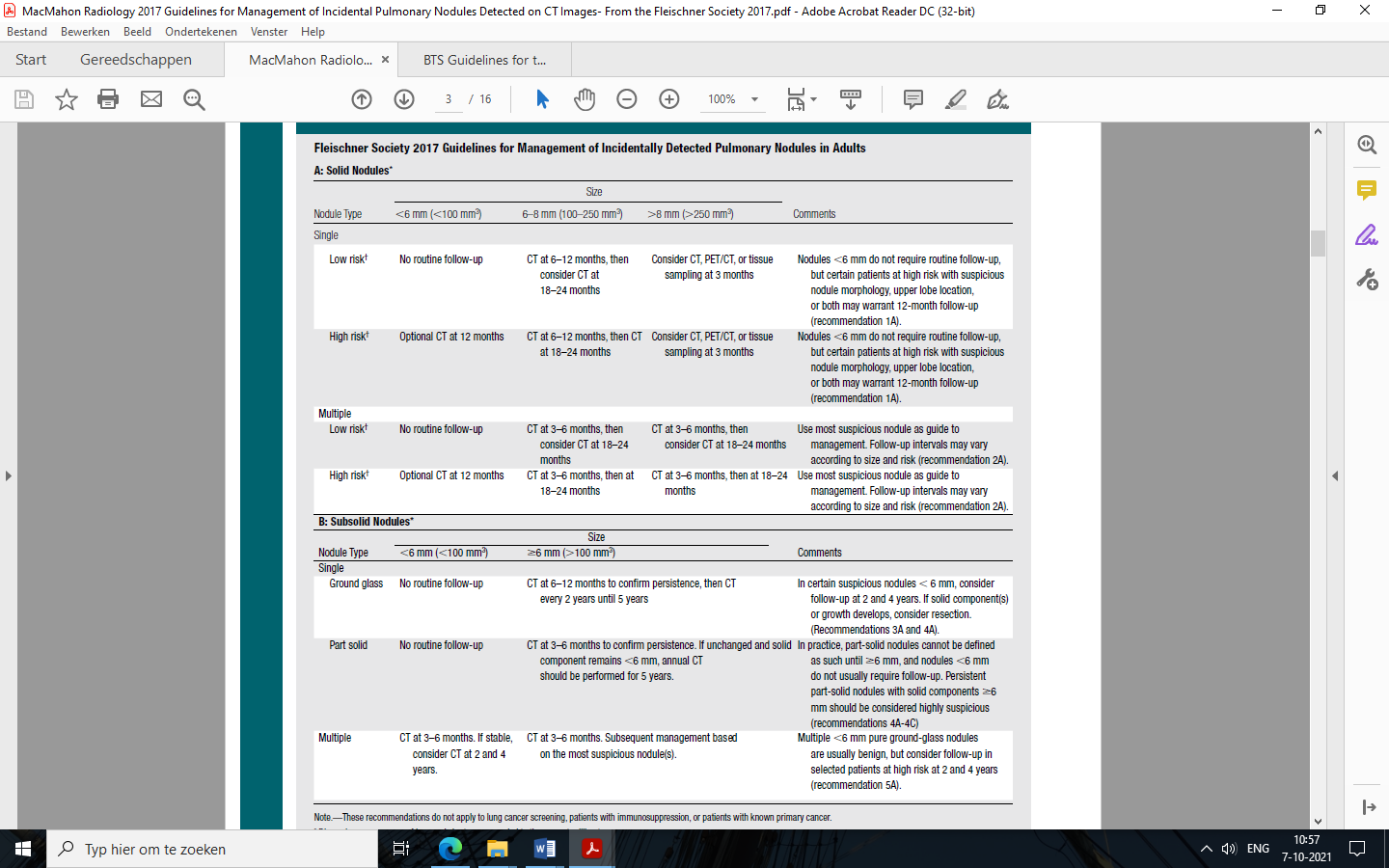


*Dimensions are average of long and short axes, rounded to the nearest millimeter.

ꝉ Consider all relevant risk factors

## References

1 Schreuder A, Jacobs C, Scholten ET, van Ginneken B, Schaefer-Prokop CM, Prokop M (2020) Typical CT Features of Intrapulmonary Lymph Nodes: A Review. Radiol Cardiothorac Imaging 2:e190159. https://doi.org/10.1148/ryct.2020190159

2 MacMahon H, Naidich DP, Goo JM et al (2017) Guidelines for management of incidental pulmonary nodules detected on CT Images: From the Fleischner Society 2017. Radiology 284:228-243. <https://doi.org/10.1148/radiol.2017161659>

## AI use disclosure

The translation from Dutch to English of this step-by-step plan is assisted by Chat GPT 4 online © 2024, the corresponding author has checked and corrected the translation if needed.
